# Extracellular vesicles and their cells of origin: Open issues in autoimmune diseases

**DOI:** 10.3389/fimmu.2023.1090416

**Published:** 2023-03-08

**Authors:** Azadeh Haghighitalab, Massimo Dominici, Maryam M. Matin, Faezeh Shekari, Majid Ebrahimi Warkiani, Rebecca Lim, Naghmeh Ahmadiankia, Mahdi Mirahmadi, Ahmad Reza Bahrami, Hamid Reza Bidkhori

**Affiliations:** ^1^ Department of Biology, Faculty of Science, Ferdowsi University of Mashhad, Mashhad, Iran; ^2^ Stem Cells and Regenerative Medicine Research Group, Academic Center for Education, Culture and Research (ACECR)-Khorasan Razavi, Mashhad, Iran; ^3^ Department of Medical and Surgical Sciences for Children & Adults, University Hospital of Modena, Modena, Italy; ^4^ Novel Diagnostics and Therapeutics Research Group, Institute of Biotechnology, Ferdowsi University of Mashhad, Mashhad, Iran; ^5^ Department of Stem Cells and Developmental Biology, Cell Science Research Center, Royan Institute for Stem Cell Biology and Technology, ACECR, Tehran, Iran; ^6^ Advanced Therapy Medicinal Product Technology Development Center (ATMP-TDC), Cell Sciences Research Center, Royan Institute for Stem Cell Biology and Technology, ACECR, Tehran, Iran; ^7^ School of Biomedical Engineering, University of Technology Sydney, Sydney, NSW, Australia; ^8^ Department of Obstetrics and Gynaecology, Monash University, Clayton VIC, Australia; ^9^ Cancer Prevention Research Center, Shahroud University of Medical Sciences, Shahroud, Iran; ^10^ Industrial Biotechnology Research Group, Institute of Biotechnology, Ferdowsi University of Mashhad, Mashhad, Iran; ^11^ Blood Borne Infections Research Center, Academic Center for Education, Culture and Research (ACECR)-Khorasan Razavi, Mashhad, Iran

**Keywords:** autoimmune diseases (AIDs), immunological tolerance and regulation, cellular therapy, extracellular vesicles (EVs), mesenchymal stem/stromal cells (MSCs)

## Abstract

The conventional therapeutic approaches to treat autoimmune diseases through suppressing the immune system, such as steroidal and non-steroidal anti-inflammatory drugs, are not adequately practical. Moreover, these regimens are associated with considerable complications. Designing tolerogenic therapeutic strategies based on stem cells, immune cells, and their extracellular vesicles (EVs) seems to open a promising path to managing autoimmune diseases’ vast burden. Mesenchymal stem/stromal cells (MSCs), dendritic cells, and regulatory T cells (Tregs) are the main cell types applied to restore a tolerogenic immune status; MSCs play a more beneficial role due to their amenable properties and extensive cross-talks with different immune cells. With existing concerns about the employment of cells, new cell-free therapeutic paradigms, such as EV-based therapies, are gaining attention in this field. Additionally, EVs’ unique properties have made them to be known as smart immunomodulators and are considered as a potential substitute for cell therapy. This review provides an overview of the advantages and disadvantages of cell-based and EV-based methods for treating autoimmune diseases. The study also presents an outlook on the future of EVs to be implemented in clinics for autoimmune patients.

## 1 Introduction

An ever-increasing number of people worldwide suffer from some form of autoimmune disease. These are clinical conditions collectively defined by the loss of tolerance/immunological inertness (1, 2), which is mainly accompanied by a simultaneous failure of regenerative mechanisms (1, 3). Losing central or peripheral tolerance could be resulted from genetic mutations, environmental factors, and stochastic or epigenetic phenomena, with possible devastating immunological consequences (4–6). Upon the activation of innate immune responses, adaptive immune responses are also triggered, which are significantly involved in determining the “extension” and “persistence” of autoimmune diseases (7). Furthermore, it was proposed that abnormal inflammatory responses are correlated with chronic autoimmune diseases (8). Activated T and B cells, inappropriately responding to self-antigens, are responsible for cell death during autoimmune diseases (1, 3).

According to some epidemiological studies, autoimmune diseases affect approximately 5–8% of the world population (9). They are associated with 80 chronic conditions (10). Autoimmune diseases are classified as either organ-specific or systemic (11). Autoimmune diseases could be considered monogenic, polygenic, or mixed patterns and may originate from deregulations in innate and adaptive immune responses (12, 13). It is unclear whether these classifications will affect the treatment strategy or outcomes, especially in cell-based therapeutics. However, these features could be considered critical in deciding which cell source or the administration route would be more suitable.

Immunosuppression is among the most common therapeutic strategies to manage autoimmunity. However, the main barrier in this approach is the complexity of the immune system and its exact fine-tuning under different circumstances. Although over-activity of the immune system is not desirable, some level of function is essential for efficient defence against pathogens. Hence, a broad-spectrum of the immune suppressants are undesirable as their long-term use may result in serious side effects ranging from susceptibility to infections to malignancies (11). Drug toxicity and low penetration capacity are other obstacles limiting the efficacy of routine therapeutic strategies (14, 15). Some pharmaceutical and even biological strategies, applied to manage autoimmune diseases in a target-specific mode, were reported to trigger new autoimmune diseases and have significant side effects (16). As a general trend, despite the cancer cases in which immunostimulatory features are desirable to control immune-scape mechanisms, in regenerative medicine, immunosuppressive features are the primary therapeutic modality to manage exhaustive tissue inflammation for the benefit of fundamental regenerative mechanisms (17, 18).

## 2 Cell-based therapies for autoimmune diseases

**Figure 1:**
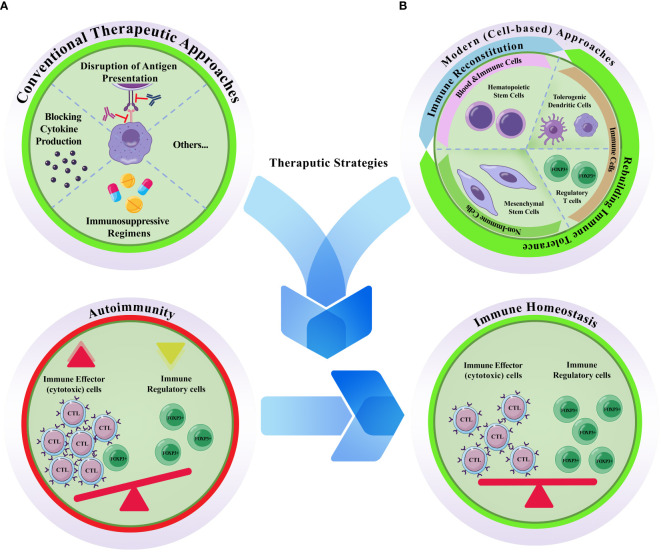
Conventional vs. modern cell based therapeutic approaches for autoimmune diseases. **(A)** Conventional therapeutic approaches are mostly depend on disruption of antigen presentation, blocking cytokine production, and/or the prescription of immunosuppressive regimens. **(B)** Modern cell-based therapeutics. Hematopoietic stem cells, tolerogenic dendritic cells, regulatory T cells, and mesenchymal stem cells are the four main cellular sources that have been proposed to treat autoimmune diseases. Autoimmunity is accompanied by abnormal accumulation of activated immune effector cells (mostly cytotoxic T lymphocytes; CTLs) and concurrent decrease/functional deficiency of the inhibitory/regulatory cells (FOXP3+) of the immune system; otherwise it is defined as the failure of the self-tolerance. Hence, effective therapeutic strategies are those with the capacity to re-induce the homeostatic state of the immune system. While hematopoietic stem cell transplantation can reconstitute the immune system, the effects of the other three cell types are attributed to their capacity for rebuilding autoimmune tolerance. Unlike the conventional methods which are typically temporary, and are accompanied by harsh side effects, cellular toxicity, and single-effect mechanism of action, cell-based methods are provide the patients with a long-lasting alleviative effects.

Cell-based therapies have been delegated to overcome the limits of conventional medicine in managing autoimmune disorders, perhaps because they affect cell-cell interactions as fundamental players of the immune responses. Understanding the molecular mechanisms and signaling pathways responsible for the pathogenesis of autoimmune diseases has provided a basis for employing cells with diverse abilities to compensate for various functional defects of the immune system. The current scope of cell-based therapies for managing autoimmune diseases has been previously discussed (19). An overview of clinical trials registered in ClinicalTrials.gov for leading prevalent autoimmune diseases indicates that four main cell types have been assessed. These include ***i***) hematopoietic stem cells (± immune ablation); ***ii***) mesenchymal stem/stromal cells (bone marrow, adipose, umbilical cord, placenta, Wharton’s Jelly); ***iii***) tolerogenic dendritic cells (TolDCs), and ***iv***) T regulatory cells (Treg) (**Figure 1**). Other cell sources/products were also reported, such as peripheral blood CD34^+^ stem cells, allogeneic lymphocytes, bone marrow aspirates, lipo-aspirates, adipose stem cells secretome, MSC conditioned media, the stromal vascular fraction (SVF) cells/vascular fraction from the adipose tissue, adipose-derived regenerative cells, autologous centrifuged adipose tissue, mesenchymal trophic factors (MTFs) from the umbilical cord, autologous apoptotic cells, and third party antigen-specific T-cells (ClinicalTrials.gov). A closer look shows that in addition to the four main cell types, some tissue components or cell suspension combinations have also been tested, containing one of these cell types or their derivatives. Different administration routes and the autologous vs. allogeneic application of the cells are other variations observed among previously registered clinical trials. First, it is necessary to highlight how much these disorders threaten global health to better describe the significance of cells and their derivatives in managing autoimmune diseases.

### 2.1 Hematopoietic stem cells

Hematopoietic stem cell transplantation (HSCT) is one of the primary efficient cell-based therapies utilized to treat autoimmune diseases (20, 21). They operate based on eliminating autoreactive immune cells and reconstituting the self-tolerant immune system. Although HSCT is an intensive one-off procedure, it brings a treatment-free remission for the patients upon success. With more than 3300 auto-immune patients registered for HSCT, the European Society for Blood and Marrow Transplantation (EBMT) Autoimmune Diseases Working Party (ADWP) has made significant progress in the field (22, 23). HSCT is currently the therapeutic modality for multiple sclerosis, systemic sclerosis, and Crohn’s disease by the professional stem cell transplant societies of America, Europe, and Brazil (24). The rationale and mode of action of HSCT were reviewed by another group (22). Given the prerequisites of this type of treatment, allogeneic HSCT is still considered a high-risk strategy that is only prescribed for patients in the very severe stages of autoimmune diseases (19, 25–27).

### 2.2 Tolerogenic DCs

Dendritic cells (DCs) are antigen-presenting cells (APCs) that regulate innate and acquired immune systems, balancing antigen-specific immunity and promoting immune tolerance. Based on their subtypes, DCs can play different and sometimes opposite roles. DCs act as pro- or anti-inflammation inducers upon external application. For instance, in the context of autoimmune diseases, they can contribute to the worsening of the disease through the production of pro-inflammatory cytokines and the activation of autologous antigen-reactive T cells (28). In the context of organ transplantation, donor DC-derived EVs can promote undesirable allograft-targeting immune responses (29). At the same time, tolerogenic DCs comprise a specific type of APCs with immunoregulatory function (30, 31).

The function of DCs depends on several factors, such as pattern recognition receptors (PPRs), specific cytokines and metabolites, some TLRs agonists, dexamethasone, and vitamin A or vitamin D3, which could be applied to maintain tolerogenic DCs stable and to enhance their lymph node-homing capacity (32, 33). The situation seems to be fine-tuned by their maturation status. Studies show that immature and semi-mature DCs demonstrate tolerogenic features (34) through different mechanisms (35). In experimental models, immature DC-derived EVs that carry tolerogenic content were applied successfully in the context of rheumatoid arthritis (36–38). DC-derived EVs maintain their producer cells’ features. Hence they could be considered proper cell replacements (39). These EVs may be enriched with either immunoregulatory or immunostimulatory content to target a particular subset of the immune cells, resulting in their suppression, reprogramming or activation. As well-known anti-inflammatory molecules, EVs with tailored anti-inflammatory content are shown to be produced by DCs, modified for the benefit of IDO1, TGF-β1, IL-10, IL-4, Fas-L, and CTLA-4 (40).

### 2.3 Regulatory T cells

Regulatory T cells (Tregs) actively maintain peripheral immune tolerance (41). Two major types of Treg cells (polyclonal and antigen-specific Tregs) have been investigated based on their immunomodulatory capacities. Pre-clinical studies indicate that antigen-specific Tregs have superior tolerogenic properties compared to polyclonal Tregs as they have more defined specificity (42). Potential applications, large-scale production, safety and efficacy of Tregs produced *via* good manufacturing practice (GMP) were recently described (43, 44).

An alternative approach to deploying tailored Tregs is *via* their genetic engineering, known as chimeric antigen receptors (CAR)-Tregs. Tregs are engineered as an improved version of the cells, which facilitates the design of advanced therapeutic procedures to induce immune-tolerogenic responses (43, 45, 46). The biology of Treg cells, advantages, challenges, and some technical details were discussed by Raffin and Bluestone (47). The possible role of these cells in effectively managing autoimmune diseases remains to be elucidated.

### 2.4 Mesenchymal stem/stromal cells

MSCs possess high regenerative capacities and special immunoregulatory functions affecting all types of innate and adaptive immune cells (48). MSCs, trans-differentiated to a macrophage-like profile *in vivo*, were shown to carry secretory capabilities that resembled phagosomes. This functional shift in the injury site is accompanied by the overexpression of CD45 and major histocompatibility complex (MHC) class II while maintaining their MSC-specific surface marker expression (49).

Due to their unique features, MSCs are currently widely applied in the late phases of clinical trials. Previous clinical studies regarding the application of MSCs for immune-related diseases are classified based on criteria, including disease type, donors, and tissue sources (50). A brief timeline for significant events in studying the immunosuppressive effects of MSCs and the progress in their clinical applications was discussed in detail by Wu et al., confirming their safety and efficacy (51).

The consequences of autologous/allogeneic MSC therapy are heavily investigated in clinical trials to treat autoimmune and inflammatory diseases along with transplant rejection, including graft versus host disease (GvHD), acute respiratory distress syndrome (ARDS), acute lung injury (ALI), ankylosing spondylitis, autoimmune hepatitis, type 1 and 2 diabetes, refractory autoimmune thrombocytopenia, skin diseases, inflammatory bowel disease (IBD), MS, CD, Stevens-Johnson syndrome (SJS), systemic lupus erythematosus (SLE), rheumatoid arthritis (RA), osteoarthritis (OA), and even in coronavirus disease of 2019 (COVID-19) as an immune-dysregulating infectious disease. As dysfunctional MSCs were proposed to be involved in the pathogenesis of some autoimmune diseases, such as SLE or immune thrombocytopenia (ITP), the utilization of MSC-based therapeutics could be considered more seriously as an effective treatment strategy (52–55).

In addition to the intrinsic capacity for migration towards inflammatory tissues, MSCs possess different immunomodulatory properties, including the inhibition of apoptosis, induction/maintenance of immune tolerance, and immunosuppression (10). The beneficial properties of MSCs, which play a role in modulating inflammatory responses and infiltration processes, have been attributed to a synergy by ***i***) MSC-released signaling molecules, ***ii***) the reaction of immune cells and other target cells to these signaling molecules, and ***iii***) feedback in the MSC-molecule-target cell axis (56). However, the precise way MSCs act is still under investigation.

MSCs produce and release extracellular vesicles (EVs) enriched with immunomodulatory factors as long as their cellular resources are kept in proper and defined conditions (57). Furthermore, they can induce tolerogenic Treg cells and even tolerogenic DCs following their administration (58, 59). The immunomodulatory, pro-angiogenic, and tissue-tropic activities of MSCs-secretome and -EVs are comparable to the producer cells, and they were proposed as valuable sources for treating inflammatory disease (60, 61). Hence, based on their paracrine effects, the potential to alter the function of immune cells, and considerable potential for EV production, we focus on these cells and their EV counterparts. The potential of MSCs for large-scale production of EVs with immunomodulatory content was compared with HEK293, ESCs, iPSCs, and ESC/iPSC-derived MSCs from different aspects in a previous publication (62). We highlight some open issues regarding the differences between cells and EVs to compare their applications.

## 3 Extracellular vesicles, a new therapeutic paradigm for autoimmune diseases

The application of cell-free products secreted by the stem and progenitor cells has been proposed to lower the risks of direct cell injection while maintaining good efficacy (63). Among several cell-free products, EVs are gaining closer attention as novel anti-inflammatory therapeutics (64). These vesicles are involved in key innate and adaptive immunity processes, including but not limited to antigen presentation, inflammation, anti-microbial defence, development and activation of B- and T-cells, and allergic, autoimmune or anti-tumor responses (65). EVs have raised a good deal of promise for the efficient treatment of autoimmune diseases, as they carry the analogous advantages of their producer cells (66). While EVs from CD3+, activated CD8+, or engineered T-cells, natural killer cells, and M1 macrophages are mostly considered immune-active nano-vesicles, EVs from regulatory T-cells, MSCs, and M2 macrophages, as well as erythrocytes, neutrophils, platelets or cancerous cells are reported to have immunosuppressive features. The key consideration is that they exert immunosuppressive or immune-active properties in a producer- and target-cell-dependent manner (67).

### 3.1 The biological properties of EVs

EVs of 30 nm to 1 µm in diameter are described as “heterogeneous bilayer membranous vesicles lacking a functional nucleus” and as “multi-signal messengers” which are enriched with a variety of biomolecules (68, 69). The biogenesis, intracellular trafficking, and secretion of EVs are tightly regulated by specific proteins, including the RAB family of small GTPases (70) and lipids, and need the molecular motors- (myosin, kinases, dynein, and GTPases) mediated cytoskeleton rearrangement (68, 71). These vesicles share characteristics regarding their membrane organizers, lipid content, cell adhesion molecules, intracellular trafficking mediators, enzymes, signal transduction, biogenesis factors, chaperones, and nucleic acids, while they are different in some others (72). Identifying these commonalities and differences between EV types will help to better understand their biology and function.

**Figure 2:**
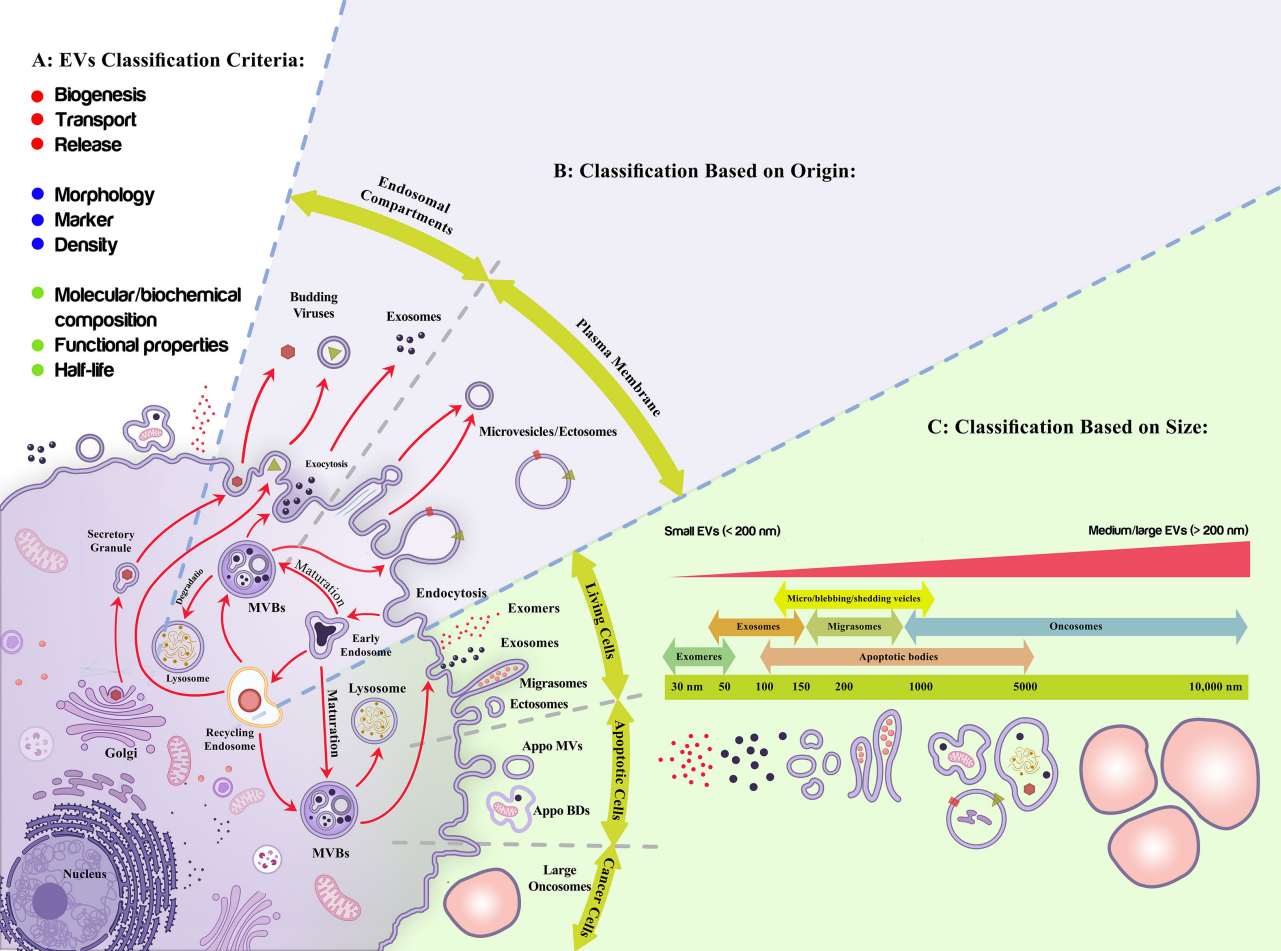
Extracellular vesicles classification. **(A)** Different criteria have been proposed to classify Extracellular vesicles (EVs) including production mechanism (biogenesis, transport, release), morphology, markers, density, half-life, molecular composition, and functional properties; among which their origin **(B)**, and size **(C)** are more applicable due to their quantitative modality and functional consequences, respectively. **(B)** As it is shown, exosomes are the nano-entities released upon the exocytosis of multivesicular bodies while ectosomes/microvesicles are produced upon the assembly of plasma membrane into the membranous vesicles. **(C)** Live cells, apoptotic cells, and cancerous cells produce nano-size extracellular vesicles with different characteristics. These vesicles have an overlapping size distribution, gradually increased from exomeres to oncosomes. EVs are also classified as small (<200 nm) and large EVs (>200 nm).

Different classification criteria and nomenclature have been applied to define various types of vesicles (73), including exosomes, microvesicles/ectosomes, apoptotic bodies, migrasomes, and oncosomes that are released by the cells under normal physiological and pathological conditions (74–76), amongst exosomes are the most well-known cellular nano-vesicles (77)(**Figure 2**). The most recently introduced member of the secreted nano-particles family includes exomeres [with a dot-shaped morphology], which are smaller than 50 nm and are enriched with metabolic pathways [glycolysis and mTORC1 signaling regulatory proteins such as β-galactoside α2, 6-sialyltransferase 1 (ST6Gal-I) and the EGFR ligand, amphiregulin (AREG) (76, 78, 79). Supermeres are the other new class of extracellular particles, indicating a different protein and RNA cargo than EVs and exomeres. Although similar to EVs and exomeres, they have the potential to carry extracellular RNA in a protected mode and play a role during chronic disease conditions (80–82). It was proposed that supermeres may be functionally involved in the immune supervision of cell-death-derived ribonucleoprotein complexes (83).

However, the isolation steps or the characterization methods for a definite type of vesicles are not convincing enough. For instance, essential controls for the different subtypes are not considered while comparing the functional immune-related properties. On the other hand, it was shown by experimental studies that their immunomodulatory characteristic is prominently diminished upon removing EVs from MSC-secretome. Although several subtypes of nano-vesicles are currently recognized, due to the recent bibliographic studies (2002-2021), two terms, “exosomes” and “extracellular vesicles”, are yet the most frequent keywords applied to describe the role of these cell-derived membranous nano-vesicles in autoimmune diseases (84, 85). Despite the initial trend, it is now evidenced that the impact of EV subtypes and their versatility is so critical compared to what it inferred at first (86, 87) and should not be underestimated during designing and performing future pre-clinical experiments and clinical trials. Here, following the International Society for Extracellular Vesicles (ISEV) consensus recommendation (88), we apply the term “extracellular vesicles” as the representative for the nano-sized natural and non-replicative particles focusing on membranous exosomes and ectosomes, which are produced by almost all cell types (68, 76, 89–92).

Isolation (93), characterization (94), labeling, and quantification strategies of different EV subtypes are constantly evolving (95). It should be noted that the various isolation methods of EVs end with different characteristics and potencies in terms of application (96, 97). It was shown that EVs from 3D cultures are functionally different and are more similar to the patient-derived EVs compared to those obtained from traditional 2D-culture conditions (98–100). These imply that spending time for optimization of isolation method/s (101, 102) to reach pure EVs with higher efficiency (103) in a reproducible manner (104) is of great importance. This way, it would be possible to compare the results more accurately (102).

EVs’ biogenesis, biological properties, and contents have been comprehensively discussed over the last few years and shown that they are essential for their function and clinical applications (68). The quality of endogenous EV preparations (105), from body fluids, tissues, or intracellular spaces (40), is affected by the circadian clock (106). In addition, isolation method, purity, storage protocol (107), time and temperature (108), presence or absence of anti-coagulants, isotonic buffers, other reagents such as cryoprotectants (109), pH, and ionic strength (110–112) are the other influencing factors. The concentration and content of EVs are influenced by demographic factors, including sex, age and race to some extent (113) and more than that, the EV cargo could be deliberately engineered through microenvironment modifications (114).

EVs are considered a powerful means for short- and long-distance communications (juxtacrine, autocrine, paracrine, and endocrine modes (65)) of the cells (115–117). These cross-talks have remained conserved throughout evolution and play a role in “inter-kingdom” mutual communications (118). This way, EVs can mediate (attenuate or promote) cell death, inflammation, and immune responses in a context-dependent mode (119, 120). Extracellular matrix-bound EVs are a recently-introduced subset of EVs with predicted roles in tissue repair and regeneration, which may be mediated by their active or passive tissue traversing or extracellular matrix (ECM) remodeling capacity (121). While cells are changing entities, EVs do not change following their release. Their biological function could, however, be impaired upon exposure to hostile microenvironments (122). In other words, EVs are membranous packages that carry various DNA-, RNA-, protein-, lipid cargoes and metabolites to their target cells (69, 123–125). They transfer active components to their target cells *via* different mechanisms, including receptors (integrins, tetraspanins, lectins, proteoglycans)-mediated binding, plasma membrane fusion, macropinocytosis, and lipid raft-mediated endocytosis, clathrin-mediated endocytosis, phagocytosis, and caveolae-dependent endocytosis, and trigger certain downstream events.

Tissue-derived EVs (Ti-EVs) reflect the tissue of origin microenvironment and cell-to-cell intercommunications and are critically important in studying and monitoring some developmental events. Like cell-culture-derived EVs, optimized and standardized protocols should be applied for efficient isolation and enrichment of Ti-EVs (126). Examples of the importance and usability of Ti-EVs have been reported in the pathogenesis, diagnosis, and treatment of Alzheimer’s disease (127, 128). EVs from individuals diagnosed with AD possess a specific protein content and miRNAs profiles which could be applied for early detection of the disease. In a recent experiment, NMDAR2A was identified as a CNS-specific EV surface marker, and it was shown in a multicenter study, including both discovery and validation cohorts, that the number of plasma EVs with NMDAR2A and L1CAM expression is lower in patients with AD in comparison to the healthy controls (129). On the other hand, as EVs are responsible for the “spread of tau pathology”, their secretion inhibition could be proposed as a novel targeted therapeutic strategy for patients with Alzheimer’s disease (126, 130, 131). Bone marrow MSC-EVs can decrease BACE-1 and Aβ, increase sphingosine-1-phosphate and exert amenable results in the context of Alzheimer’s disease (66, 132, 133).

Preparing qualified endogenous EVs from bio-fluids as the primary source is more complex, as several factors interfere with efficient EV isolation and purification (134). The quality of the final sample will be determined as a consequence of donor-related factors in addition to the biophysical and chemical properties of the fluid. The material of the sample collection tube, the application of coagulant or anticoagulants, the initial volume of the sample, mixing or agitation steps, the duration of pre-processing or pre-storage steps, and hemolysis degree or other depletion strategies are among the essential determinants of the quality of EVs. It is also essential to manage possible contaminations (111, 135).

Among their broad applications, EVs are sometimes utilized based on their nano-sized features and ability to pass through blood barriers (136). In such cases, EVs could be applied as a new paradigm with amenable priorities (137, 138). EVs can achieve most features of the synthetic nano-carriers (i.e., liposomes and nano-particles). Moreover, they have higher biocompatibility, chemical stability, longer-distance intercellular communications, and cell fusion capacity (139). As non-toxic nano-carriers, EVs demonstrated better pharmacokinetics and pharmacodynamics features (absorption, distribution, metabolism, and excretion) than synthetic nano-carriers (140, 141). However, there are controversies regarding their application as drug delivery systems (DDSs) like nano-shuttles (142). Some technical limitations have been raised regarding their flexibility in applying different reagents, preparation procedures, and surface functionalization strategies (139). Also, they have widely been recognized as natural and valuable means for diagnosing and monitoring various disease conditions and novel therapeutic agents (111, 143).

### 3.2 Therapeutic potential of EVs

Therapeutic applications of EVs in managing different inflammatory and autoimmune diseases and their immunomodulatory features and composition were discussed in recently published articles (144–146). Extensive parallel research during the last decade has revealed new aspects in the biology of MSCs, leading to changes in our orientation regarding their applications. These changes highlighted the importance of the paracrine effects of MSCs and proposed the possibility of replacing the cells with their cell-free products, especially in the allogeneic context (147). “Secretome” is the common term that represents all substances released by MSCs, including free-soluble factors (various growth factors, chemokines, and cytokines) and insoluble EVs (148–150). Different aspects of the pleiotropic therapeutic effects of MSCs and their paracrine action were discussed in a recent publication, emphasizing the active components of the MSCs’ secretome (116). The paracrine strength of these cells is so significant that it has been suggested that the name of these cells would be changed to the medicinal signaling cells (151, 152). Munoz-Perez et al. reviewed the latest trends regarding the potential of MSC-secretome to treat immune-encountered inflammatory diseases (153).

There is a growing tendency toward local administration of EVs employing diverse scaffolds from acellular tissues to solid patches and hydrogels (154–158). One good reason favoring the local administration of EVs is the lower amount required, making their utilization much more cost-effective. Furthermore, their gradual and sustained release following the administration is a positive point (87). Moreover, the application of scaffolds/biomaterials increases the bioavailability, homing, and/or biocompatibility of EVs at the site of injection, while they are also effective in avoiding the side effects of the injection procedure (159). This preference may be pronounced when dealing with local inflammatory conditions or tissue-specific autoimmune diseases.

It was confirmed that MSC-EVs are therapeutically efficient in 30 animal models mimicking various human diseases (111). While different roles were described for EVs during the pathogenesis/immunopathology of Rheumatoid arthritis and joint impairment of RA patients, the therapeutic impact of MSCs- and non-MSCs-EVs (dendritic cells, polymorphonuclear neutrophils, myeloid-derived suppressor cells) were confirmed in animal studies (160). The first case report regarding the benefits of the MSC-EVs was related to a patient with GvHD (161). The high significance of MSC-EVs, as a novel therapeutic for managing Alzheimer’s disease, has been thoroughly discussed recently in mice and human cases, emphasizing the mechanisms of the disease progression (66).

Similar to their producer cells, MSC-EVs eventually found their place in pre-clinical and clinical studies (162). However, each step of scalable EV manufacturing under GMP compliance is still exposed to several challenges (163, 164). These challenges exist not only in upstream and downstream stages (165, 166) of EV production and isolation, such as cell culture and purification steps, respectively, but also during EV storage (108), quality control (167), and functional evaluation steps (148, 168).

EVs were previously enriched from the conditioned media of different immune cells, including dendritic cells, T regulatory cells, M1/M2 macrophages, and CD4^+^ T cells (169–171), and their potential was evaluated in the context of autoimmune diseases (44). However, despite the initial trend, it is now evident that these vesicles, regardless of their producer cell type, are produced with different biological content under different circumstances (172), and their application is accompanied by several challenges and complications (173–175). One remaining question could be: How can we learn from the long-term and extensive research on their producer cells to make EVs-based therapeutics safe and efficient in the frame of translational medicine? Finally, the main questions considered to be addressed by this review are the level of superiority of EVs to intact cells in reducing patients’ symptoms of autoimmune disorders and the maturity of techniques for preparing clinical-grade EVs (176).

## 4 MSC-EVs vs. MSCs: Open issues

### 4.1 Interaction with the immune system

MSCs have immunosuppressive and immune privilege properties; hence, their interactions with the immune system are expected to benefit immune homeostasis and restore immune tolerance. However, there is concern regarding their allogeneic and repeated administration (177). MSC-EVs, like their original cell sources (MSCs), are believed to have low immunogenicity as well as lower carcinogenic risk (178). They do not release certain inflammatory factors (IL-1, IL-6, and IL-8), which may be vastly released from dead cells following cell injection procedures, especially in tissues lacking adequate blood/nutrients (179). In addition, EVs demonstrate enhanced circulation stability due to the immune system’s evasion (39). Although similar to allogeneic cells, these particles may potentially carry some immunogenic proteins (e.g., MHC molecules), it is open to question whether these proteins are transferred from the vesicles to other cells in communication and if they can trigger alloimmune responses (**Table 1**) (29, 177, 180).

**Table 1:** EVs have differences in comparison to their producer cells.

Open issues		Significant differences
**Interaction with the immune system**	**Stability**	- More stable in the circulation (39).
	**Allogeneic Administration**	- Carry immunogenic proteins with questionable ability for transferring the proteins to the recipient cells (29, 177, 180). - The immune rejection rate of EVs is considerably low compared to the cells (181).
	**Heterogeneity**	- Heterogeneous temporal, spatial, or disease-associated nature. - Have more common features in comparison to the cells.
	**Mechanism of** **Tolerance Induction**	- The way EVs communicate with the effector cells of the immune cells is incompletely described (182). - EVs can penetrate deeper into the damaged tissues, so they may have a higher chance of regulating the immune cells (183). - EVs have less cell-dependent and independent mechanisms to exert their immunomodulatory properties (183–187). - EVs immunoregulatory properties leading to reduced immune reactions, increased tolerance, and homeostasis (188, 189). - EVs have been shown to carry, mediate, and regulate cytokine transport from cells to members of the immune system (190). - Allogeneic EVs do not stimulate T cells during *in vitro* experiments unless in the presence of APCs. - MSC-EVs cannot impact the production of CD3^+^ T cells. They do not change the ratio of B cells and plasma cells *in vitro* (182, 191–193). - MSC-EVs inhibit the complement activation in a CD59-mediated manner (194). - EVs are less functional against DCs in comparison to the cells (185, 195). - Allogeneic EVs would activate T cells *in vivo* only if delivered into an inflammatory microenvironment (196).
**Homing capabilities**	**Systemic Administration**	- Proteins and glycoproteins on the surface of EVs and the recipient cells mediate EV-cell interactions. - EVs interact with recipient cells through various targeting mechanisms to transmit selective biological information. - Superficial cytokines on EVs could act as barcodes recognized by the recipient cell cytokine receptors (197). - On the contrary, some studies suggest that EV uptake is not cell-specific (198).
	**Uptake Mechanism**	- The interactions between EVs and the neighboring or distant acceptor cells occur through diverse mechanisms (115). - It is proposed that MSC-EVs have the same receptors of MSCs on their membrane, and that could be why MSC-EVs could find the injury site, perhaps through a similar mechanism (199). - EV size and surface components affect their recognition and capture by acceptor cells (115). - EVs content could randomly be released into the cytoplasm upon fusing vesicles with the cell membrane. - Acceptor cells may internalize vesicles to intracellular specific molecular targets.
	**Communication with the Environment**	- Although different from their producer cells, EVs also alter the microenvironment. - It is possible for EVs to acquire soluble proteins from the surrounding extracellular milieu (200–202). - EVs harbor their cargo from enzymatic degradation while trafficking through the extracellular milieu (203). - EVs preserve normal tissues, support tumorigenesis, provide nutrition, and facilitate immune escape (204).
**Application**	**Clinically Relevant Superiorities**	- EVs mediate a significant part of the paracrine action of stem cells and most of their functional properties (139). - Unlike cells, there is no concern regarding the possibility of necrosis or abnormal differentiation (181). - They do not show self-replicative and tumor-formation properties (139). - They are not seriously affected by the surrounding inflammatory microenvironment (186). - They reach deeper into injured tissue layers (183). - EVs are highly bio-stable, hence their contents are protected from macrophage-based phagocytosis (205). - EVs have a more straightforward pre-banking capacity and are more stable during freeze-thaw cycles. - EVs can be combined with existing compositions or drug delivery methods (66).

MSCs‐EVs exert their immunomodulation through a similar mechanism known for MSCs‐associated tolerance. This process is mediated by programmed death ligand‐1, galectin‐1, and TGF-β (206). They impose immunomodulation instead of immunosuppression, leading to reduced immune reactions and homeostasis (188, 189). MSC-EVs contain many parental biomolecules, including chemokines, cytokines, and growth factors (207). Suh et al. described the mechanisms responsible for the therapeutic effects of EVs in the context of different inflammatory diseases. Similar to the cells, the mechanisms responsible for the therapeutic effects of the EVs in the context of different inflammatory diseases are reducing the microglia/macrophage activation, oxidative stress, pro-inflammatory cytokine and chemokine release, T-cell activation, tissue fibrosis, viral infectivity, immune cell infiltration, and apoptosis/necrosis (146). Moreover, EVs regulate M1/M2 macrophage polarization for the benefit of M2 cells, induce collagen regeneration, and prevent scar generation (181).

Despite similar immunomodulatory properties, differences in the two entities’ level and mechanism of action have been reported. The way EVs communicate with the effector cells of the immune cells is not as simple as it sounds and the mediators of MSC-EV-based immune modulation are yet remained incompletely described (182). In comparison to MSCs, EVs have less cell-dependent and independent mechanisms to exert their immunomodulatory properties upon exposure to various immune system cells (183–187). On the other hand, EVs can penetrate deeper into the tissues (208, 209), so they may have a higher chance of regulating the immune cells (183). In addition, the therapeutic anti-inflammatory properties of EVs could be more pronounced due to their capacity for passing through blood barriers (210). Also, the immune rejection rate of EVs is considerably low compared to the producer cells, as they have a low-level expression of membrane histocompatibility molecules (187). EVs have been shown to carry, mediate, and regulate cytokine transport from cells to members of the immune system (190).

Unlike the cells, MSC-EVs cannot impact the production of CD3+ T cells. Allogeneic EVs could bind to T cells, but they do not stimulate these cells during *in vitro* experiments unless in the presence of APCs in the culture media. In addition, EVs do not change the ratio of B cells and plasma cells following their co-culture with human peripheral blood mononuclear cells (PBMCs) *in vitro* (182, 191–193). Allogeneic EVs would activate T cells following their *in vivo* administration in mice only if delivered into an inflammatory microenvironment (196). MSC-EVs can interfere with the feed-forward loop between complements and neutrophils *via* the inhibition of the complement activation in a CD59-mediated manner. Hence, MSC-EVs are influential players able to modulate inflammatory status spatially and temporally (194). While MSCs and their cell-free counterparts can increase the ratio of FOXP3+CD25+CD4+ T regulatory cells, EVs are less functional against DCs (185, 195).

The therapeutic effects of extracellular vesicles as new biologicals for immune regulation have been supported by pre-clinical studies. Native or modified MSC-EVs were used to regulate the immune-associated cells while focusing on their mechanism of action (211, 212). There is almost no doubt that MSC-EVs can trigger anti-inflammatory responses based on restoring the balance action in the milieu or immune cells. This could be attributed to their miRNAs, other non-coding RNAs, or protein cargo; otherwise, surface EV-associated proteins may pave the role by activating the downstream intracellular pathways in recipient cells (211, 213). The heterogeneous nature of producer cells, diverse EV isolation, quantification and standardization strategies and the critical role of disease-specific pathways are considered the main challenges to introducing unique regulatory mechanisms (212, 214). Moreover, the clinical practice of EVs is challenged by their heterogeneity to some extent. Optimization of current digital assays has been proposed to do single-vesicle-resolution studies, especially during the clinical analysis of liquid biopsies (215). Immune biocompatibility of EVs is the other criterion to be investigated in autoimmune diseases. It was shown in a previous study that although both autologous and allogeneic small EVs may have efficient therapeutic effects, autologous EVs are more viable and impactful in damaged tissues (216, 217).

Recent updates unravel the crucial role of EVs and vesicular trafficking in the immune system (65) and during the pathogenesis of immune-based diseases, in addition to their therapeutic perspectives (218). An accurate understanding of the mechanisms by which tumors or other pathological statuses induce immune cell dysfunction *via* their extracellular particles will allow a better understanding of how vesicles interact with the cells of the immune system. These data would potentially facilitate developing novel methods to face the overactive immune system and the associated diseases.

### 4.2 MSC-EVs vs. MSCs: Homing capabilities

The migratory capacities of MSCs towards ischemic and damaged sites have received substantial consideration for treating diffuse and localized inflammatory and degenerative conditions (219). However, low homing efficiency is a significant drawback associated with temporary therapeutic benefits (220). Hence, diverse approaches have been employed to enhance the entrapment rate of systemically infused MSCs (221). EVs must also follow the route to the affected areas as membrane-bound cell-derived structures. Various cell-to-cell communication functions are ascribed to EVs, including autocrine and paracrine missions (222). Sung et al. discovered that EVs could even contribute to directional cell migration. They confirmed that malignant cells could find their path toward EV deposits, resulting in EVs’ endocytosis (223). In specific migration paths, cells use almost the same delivery process as depicted for growth factors and peptides, in addition to their active migration (224). Here, we raise some open issues to compare MSCs and EVs concerning their homing capabilities.

When systemically administered, exogenous MSCs imitate the delivery route *via* which endogenous MSCs reach their destination (225). As tissue injuries often coincide with inflammation or ischemia, inflammatory factors could provide cues to mobilize cells towards the damaged tissues. The expression of homing receptors on the cell surface is proven to mediate MSCs’ migration towards their ligands at the ischemic target microenvironment (226). Upon arriving at the damaged site, circulating MSCs or their bioactive components need to leave the vasculature and transmigrate across the endothelium to get into the stromal region, where they exert their primary function both in passive and active manners (220, 227). Paracrine interactions of culture-expanded cells and the target milieu necessitate reciprocating various bioactive determinants (228). Furthermore, the bio-distribution of exogenous MSCs has always been challenging, as many infused MSCs are entrapped in other organs, particularly the lungs, liver, and spleen (220).

#### 4.2.1 Are EVs capable of finding their targets too?

The capability of MSCs to desirably home in insulted tissues is of substantial cell-based treatment advantages. It has been shown that some migratory axes, such as chemokines at the site of injury and chemokine receptors on the surface of cells, namely the SDF-1/CXCR4 axis, orchestrate the stem cells’ homing process (229, 230). Regarding the selective EV uptake, proteins and glycoproteins on the surface of EV and the recipient cell mediate EV-cell interactions. Hence, most strategies that were applied to increase the tissue specificity of EVs are trying to reconfigure the surface glycoproteins of the vesicles (231). Through various targeting mechanisms, EVs interact with recipient cells to transmit selective biological information. Superficial cytokines on EVs could act as barcodes recognized by the recipient cells’ cytokine receptors (197). On the contrary, some studies suggest that EV uptake is not cell-specific (198).

#### 4.2.2 How do the uptake process mechanism and homing, about extravasation and transmigration of vesicles, differ from that of stem cells?

Homing of stem cells is described as the arrest of cells within the vasculature of a tissue followed by transmigration across the endothelium, usually in response to a chemokine gradient in the target region. The leading role of receptor/ligand axes, e.g., VLA-4/VCAM-1, is highlighted in its contribution to tethering, rolling, firm adhesion, and transmigration of the cells (220).

EVs were reported to demonstrate cell and tissue-specific autonomous targeting capabilities (139, 215). The interactions between EVs and the neighboring or distant acceptor cells occur through diverse mechanisms. EVs’ size and surface components affect their recognition and capture by the acceptor cells (115). The literature is inconsistent on whether EVs can target specific tissues, mainly due to the diverse tracking methods. It is proposed that MSC-EVs have the same receptors of MSCs on their membrane, which could be why MSC-EVs could find the injury site, perhaps through a similar mechanism (199, 232). While some studies depict no specific *in vivo* biodistribution of EVs, others show they tend to accumulate in tumors or injured tissues, which could explain EVs’ therapeutic effects (233).

The receptor-mediated binding of EVs to cells could stimulate a signaling cascade to transmit information without delivering their content. Upon the fusion of vesicles with the cell membrane, EVs content could be randomly released into the cytoplasm. Additionally, acceptor cells may internalize vesicles to intracellular specific molecular targets. Clathrin-dependent, clathrin-independent pathways such as caveolin-mediated uptake, macropinocytosis, phagocytosis, and cholesterol-rich lipid rafts mechanisms are among the numerous endocytosis processes outlined in the literature (203).

Clathrin-mediated endocytosis requires adaptins, which connect membrane cargo to clathrin, forming a polyhedral lattice surrounding the vesicle. Clathrin-mediated endocytosis involves the assimilation of receptors based on their ligands in clathrin-coated pits on the plasma membrane, collapsing into a vesicular bud and forming clathrin-coated vesicles. The subsequent intracellular vesicle goes through clathrin uncoating and then integrates with the endosome to deposit its contents. Some treatment approaches, e.g., cancer, prevent clathrin-coated pits, resulting in decreased EVs’ uptake (222). Caveolae-mediated endocytosis evolved from the oligomerization capacity of caveolin proteins. Oligomerization of caveolins mediates the formation of caveolin-rich rafts in the plasma membrane. Caveolae are tiny cave-like introversions in the cell membrane that can become internalized into the cell (203, 222).

#### 4.2.3 How do EVs communicate with their target microenvironment?

To segregate EVs from soluble mediators, MISEV-2018 has suggested that cell-cell-contact could be regarded as necessary for signaling, but it may also happen by exchanging plasma membrane-enclosed signals between EV-donor and EV-recipient cells (91). These vesicles harbor their cargo from enzymatic degradation while trafficking through the extracellular milieu (203). EVs, are not the same as their parent cells but contribute to altering the microenvironment. EVs are shown to manage specific paracrine intercommunication in the tumor microenvironment (234). As tumor progression starts to develop, the surrounding microenvironment performs anti-tumor immunity and aims to subside tumorigenesis. Once the tumorigenesis progresses, the microenvironment evolves into tumor-conducive. Cancer cells take advantage of EVs’ paracrine intercommunication through conversion from a normal milieu to a tumor microenvironment. EVs are used to preserve normal tissues then support tumorigenesis, provide nutrition, and facilitate immune escape (204). It is possible for EVs to acquire soluble proteins, and hence new biological functions, from the surrounding extracellular milieu. The main determinants are the physical and biochemical characteristics of their surface and protein concentration-based environmental changes. For example, during the pathogenesis of autoimmune diseases and innate and acquired immune responses, autoantigens may be packaged in EVs prior to or post-release (200–202).

### 4.3 Shifting from MSCs to MSC-EVs regarding their application

Differences between cells and their extracellular descendants have put a debate regarding their potential applications (see **Table 2** for a detailed comparison). The preparation of characterized EVs and their standardization needs more interdisciplinary knowledge, as they are nano-sized entities (273). In the case of cells, there is less concern regarding the preparation of a determined number, while in the case of EVs, it is not as easy due to the differences in the availability and sensitivity of the quantification methods (274). Different criteria have been applied to quantify EVs, including the number of producer cells in the culture, the protein content of EV preparations, and the number of EVs/ml of the final preparation. This puts a big hurdle in comparing the results of different experiments (185). Unlike cells that need vascular structures to receive essential nutrients and survive, EVs could be administered in tissues with a lower capillary network, such as intervertebral discs. Also, as EVs do not release toxic or harmful metabolites, there is no concern regarding their utilization in such tissues (179).

**Table 2:** Comparison between the cells and EVs regarding their intrinsic and application properties.

Properties		Cells	Extracellular vesicles
**Intrinsic**	**Nature**	- Natural, bilayer membranes with heterogeneous & cell-dependent distribution of glycolipids and glycoproteins	- Natural or synthetic, biomimetic (235), bilayer membranes
	**Morphology**	- Highly heterogeneous	- Heterogeneous
	**Physicochemical**	- Large	- Small, higher resistance to low temperatures (185)
	**Prone to change**	- Responsive to the environment (185, 186)	- Non-responsive to the environment (185)
	**Proliferation**	- Proliferative/self-replicative (185)	- Non-proliferative (185)
	**Functional**	- Self-renewal and differentiation capacity (*in vitro*) (236) - Secrete active compounds, can initiate tumorigenesis (237) - Senescence may induce thrombosis by obstruction of small blood vessels (237)	- Mediators of intercellular signaling/communication (238–240) - Paracrine and autocrine actions on stemness maintenance or cell differentiation (241) - Exhibit producer cell-dependent phenotypes (242–244) - EVs’ cargo can be drastically altered by culture conditions (243, 245) - EVs can mediate tumor initiation, progression, angiogenesis, and metastasis (235, 246) - EVs play role as disease biomarkers (239, 247, 248) - EVs demonstrate an age-related content (249) - MSC-EVs have prothrombotic effects (237)
**Administration**	**Size**	- Because of their size (10 µm), they can obstruct capillaries	- Pass through capillaries and BBB, and spinal cord barriers (250–253)
	**Dosage**	- Their potential for proliferation can limit the dose of administration	- They can have a wide range of administered doses
	**Route**	- Intravenous, intrathecal, intraventricular, subarachnoid, intra-arterial, intraperitoneal	- Intranasal, intravenous, intraperitoneal, intracranial, intracochlear, inhalation, oral, subcutaneous (141, 253–255)
**In the body**	**Immunogenicity**	- Low risk of immune rejection	- Lower risk of immune rejection (185, 187) - EVs are involved in antigen presentation (256) - Pathogenic EVs contain autoantigens (241) - Regulate the migration, proliferation, activation, and polarization of various immune cells (257, 258) - Promote a tolerogenic immune response (259, 260) - Inhibiting inflammatory response (259, 260) - Stimulate or suppress anti-cancer immunity
	**Circulation time**	- Short circulation life	- Longer circulation life (137, 261)
	**Target cell selection**	- Homing capability to some target sites, such as ischemic tissues	- Innate tropism to specific sites, which can also be engineered (76) - Exhibit cell-targeting properties (111)
	**Long term effects**	- Limited proliferation potential	- Not clear yet
	**Tracking strategies**	- X-ray–based methods (plain films and computed tomography (CT), optical imaging (bioluminescence and fluorescence), ultrasound/echocardiography, single-photon emission computed tomography (SPECT), positron emission tomography (PET), magnetic resonance imaging (MRI) (262) - Single-cell Tracking of cells (175)	- Membrane labelling, vesicle interior labelling, labelling EV-specific cargoes (1, 69)
**Manufacturing**	**Isolation**	- Relatively simple isolation and characterization methods	- More complicated isolation steps (263)
	**Storage**	- Not possible to store them at room temperature, reduced viability after freeze and thaw	- Long-term preservation and storage stability (241)
	**Off the shelf**	- Not an off-the-shelf product	- Potent to be stored as lyophilized material (112) - Easier scale-up (185, 264, 265)
	**Biopharmaceutical**	- Biocompatible (137) - Biodistribution preference to lungs, liver, and kidneys (137) - Suitable for multi-drug delivery (137)	- Biocompatible (181) - Biodistribution preference to the liver, administration route-dependent biodistribution (266) - Untargeted accumulation in tumor tissues (137) - Intelligent Nano-carriers (267) - Multifunctional drug delivery systems (266)
**Manipulation**	**Loading methods**	- Various drug encapsulation methods (137, 268)	- Suitable manipulative platform (269) - Various exogenous and endogenous loading (205, 270) methods (235, 271, 272)
**Regulations**	**Regulatory bodies** **and international communities**	**- FDA** (Food and Drug Administration) **- ISCT** (International Society for Cell and Gene Therapy) **- ISBT** (International Society of Blood Transfusion) **- ISSCR** (International Society for Stem Cell Research)	**- FDA** (Food and Drug Administration) **- ISCT** (International Society for Cell and Gene Therapy) **- ISEV** (International Society for Extracellular Vesicles) **- ISBT** (International Society of Blood Transfusion)
	**GMP standards and guidelines**	- Well-defined GMP standards	- Lacking good manufacturing standards (137, 142) - Guidelines for novel EV-based therapeutics (111)

Having mentioned these differences, EVs generally have some clinically relevant superiorities to cell therapy (185, 264, 265). The immune rejection rate of EVs is considerably low compared to the producer cells (181), so they have an increased half-life and are more stable in circulation (39). EVs demonstrate an innate tropism to specific tissues (76) and exert cell-targeting properties (111). Unlike cells, there is no concern regarding the possibility of necrosis or their abnormal differentiation (181). They do not show self-replicative and tumor-formation properties (139) and are not seriously affected by the surrounding inflammatory microenvironment (186). They reach deeper into injured tissue layers (183). Moreover, EVs have a more straightforward pre-banking capacity, are less sensitive to low temperatures, and are more stable during freeze-thaw cycles. EVs can also be combined with existing compositions or drug delivery methods (66). These vesicles would provide the scientists with a suitable manipulative platform (269).

## 5 EVs from modified cells and EV modification strategies

EV secretion is likely affected by different pharmacologic and/or environmental insults that target producer cells’ cytoskeleton (115, 275). Furthermore, another specific priming/stimulating strategy may be applied as one of the most convenient strategies to persuade the cells to produce and secrete EVs with desirable features/contents (276–278). In a hybrid approach, to facilitate the large-scale production of EVs with determined properties, Gomzikova et al. evaluated the immunomodulatory properties of the cytochalasin B-induced membrane vesicles (CIMVs). They demonstrated that human MSC-EVs prepared with this approach could inhibit the activation and proliferation of human PBMCs (279). Yuan et al. demonstrated the beneficial consequences of a 3D dynamic culture of aggregated hMSCs for the efficient generation of 3D-hMSC-EVs regarding their size, concentration (EVs/Cell/2 days), common exosomal markers (CD63, Alix, Flotillin-2, and CD81), and immunomodulatory capacity (IDO activity) in the presence or absence of interferon-gamma (280).

EV modification strategies could also be utilized to enhance the accumulation of the particles in desirable target organs, change their surface properties, or reduce their phagocytosis and endocytosis by macrophages (62). To reduce immune cell recognition, polyethylene glycol (PEG) is a practical approach to increase the circulation time of EVs (178). It was shown that preparing engineered EVs (EEVs) with surface expression of CD47, due to their bio-inertness and immune-evasive properties (178), will provide us with vesicles that are less prone to systemic clearance (281). Engineered EVs were also innovatively equipped with receptors that adsorb pro-inflammatory cytokine IL-6 from diseased muscle tissue with chronic inflammatory status (282).

A successful example of engineered EVs as autoimmune disease therapeutics was proposed by Zampieri et al. in a pre-clinical study. To induce immune tolerance *via* molecular farming, they designed plant virus nano-particles displaying recombinant peptides associated with autoimmune diabetes and rheumatoid arthritis. They demonstrated that the virus structure could play carrier roles for the recombinant peptide and adjuvant, as tomato bushy stunt virus (TBSV) demonstrated intrinsic immunomodulatory properties. However, to a lesser extent, the viral particles carry the recombinant peptides (283).

**Figure 3:**
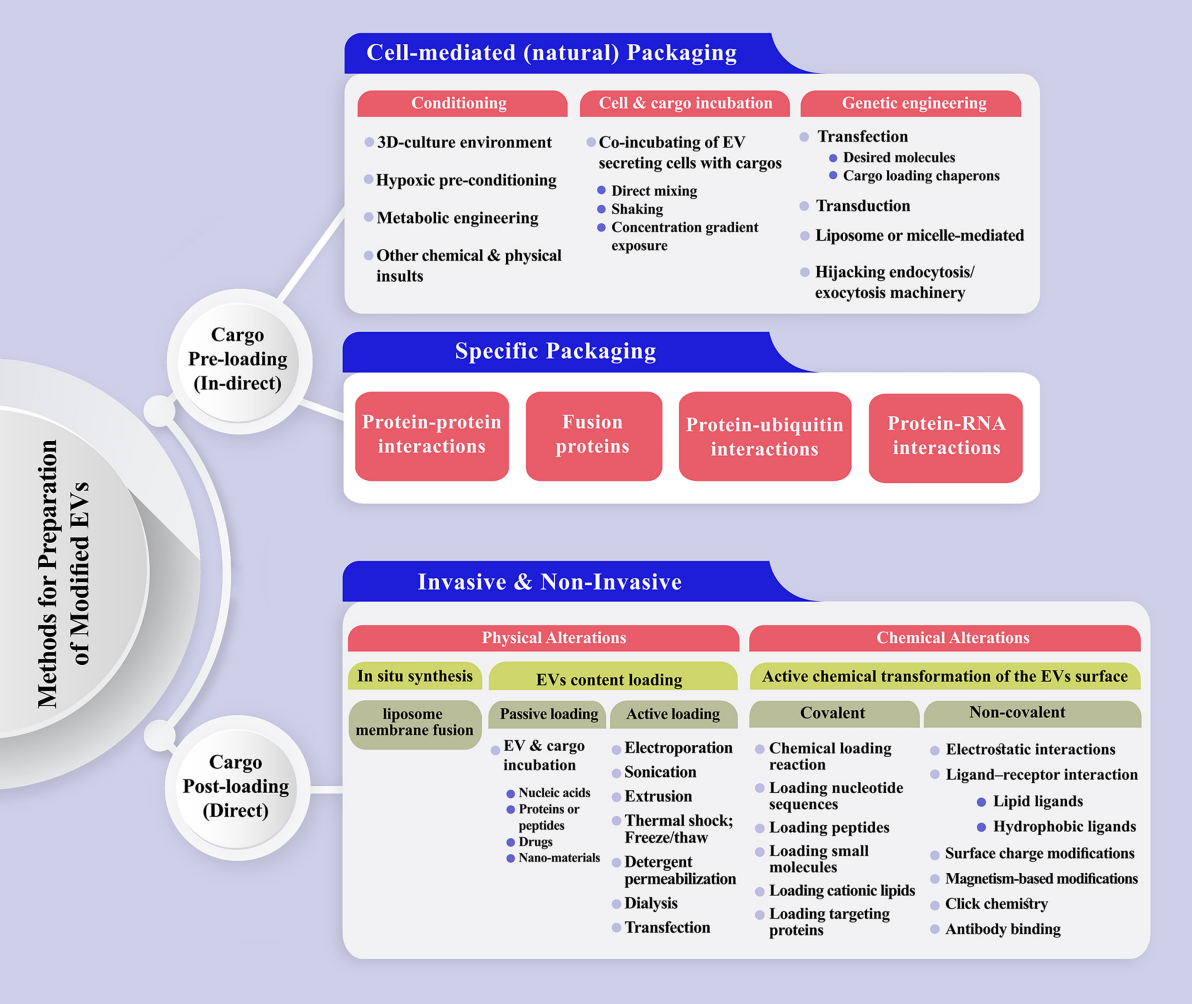
A schematic representation of the methods has been proposed to prepare engineered extracellular vesicles. (Top) Cargo pre-loading (in-direct) path is subdivided to cell-mediated and specific packaging fashion. The latter one could be designed based on protein-protein, protein-RNA, protein-ubiquitin interactions or the production of fusion proteins. (Bottom) Direct EV post-loading is achievable *via* physical and chemical modifications which are the two major ways for transferring the cargo of interest to EVs. Physical alterations mostly include passive and active loading techniques. Incubating EVs with the cargo of interest (passive loading) is a convenient and effective way, which was applied to load EVs with nucleic acids, proteins or peptides, drugs, and nano-materials. Electroporation, sonication, extrusion, thermal shock, detergent permeabilization, dialysis, and transfection are the main active loading techniques for intensifying the content of EVs in the benefit of desirable molecule/s. Covalent and non-covalent interactions are proposed as active chemistry methods to modify the inner or outer surface of EVs.

Cargo pre-loading and post-loading could be considered a primary classification for EV modification (284–286)(**Figure 3**). Natural or specific packaging could be applied to pre-load EVs with target cargo. The producer cells are loaded with the molecules of interest during natural packaging through cell conditioning, cell and cargo incubation, or common cell modification methods, such as transfection, transduction, and electroporation (124, 287). Moreover, exogenous materials could be introduced to the EVs based on liposome or micelle-mediated mechanisms. For more efficient drug delivery, EV bilayer membranes are commonly permeabilized to allow the dynamic loading of the vesicles (288, 289). In specific loading, the basis of cargo enrichment lies in protein-protein interactions, the fusion of proteins, protein-ubiquitin, and protein-RNA interactions. EV post-loading is achievable *via* physical and chemical modifications, which are the two major ways for transfering the cargo of interest to EVs. Incubating EVs with the cargo of interest (passive loading) is a convenient and effective way, which was applied to load EVs with nucleic acids, proteins or peptides, drugs, and nano-materials (205, 290, 291). Different methods are proposed for direct loading of EVs with cargos of interest, such as the application of transfection reagents, electroporation, incubation, sonication, freeze/thaw cycles, saponin, extrusion and dialysis (205, 270). The loading efficiency of EVs depends on several factors, mainly the quality of the starting material, the physical and biochemical properties of cargo, and its stability and functional maintenance during the loading process (286). Covalent and non-covalent interactions are proposed as active chemistry method subsets to modify the inner or outer surface of EVs, each of which includes a variety of methods, gradually updated based on recent innovations (292).

Three models were proposed as the primary cell-mediated packaging of EVs with desirable nucleic acids (293): induced packaging, cellular protein-assisted packaging, and engineered protein-mediated packaging. To reach therapeutic-grade EVs, they are prepared from specific producer cells (294). Otherwise, modified molecules (proteins or chemical compounds) with organ-specific tendencies are introduced to the EV surfaces (295). Various interfering mechanisms also play significant roles, including intracellular calcium level, external stress, cytoskeletal blocking, and the consequences of specific gene expression could be highlighted (124). Following the entry to target cells, EVs, enriched with coding or non-coding oligonucleotides, trigger gene expression pathways or regulate specific gene transcription procedures. However, the risk of EV internalization in the endosomal compartment, degradation, and re-release of intact vesicles should be considered (296).

Protein-loaded EVs are mainly administered as fluorescent reporters or targeting moieties (288). Regardless of the engineering or priming strategy applied to prepare modified EVs, one should consider the immunogenic potential of surface-engineered EVs. For example, tumor-targeting antigenic (TTA) peptide glycoprotein 100 (gp100) engineered EVs are rapidly internalized by APCs and are highly immunogenic for stimulating cytokine production (297). Engineered EVs were applied in pre-clinical studies and have found their way into engineered EV-based therapeutics (269, 298).

A deep molecular understanding of various biogenesis pathways and structural properties is essential for designing desirable EVs. Further, we should consider the variety of molecules that are involved in the specific sorting of various cargoes to the small and large EVs, including Ras-related (RAB) GTPases in the brain, glycosphingolipids, flotillins, chaperone HSC70, and small integral membrane proteins of the lysosome/late endosome (SIMPLE) (299–301).

Clinical translation of the EVs is affected by different issues, including isolation, purification, standardization, yield, and functional heterogeneity (287, 302). Accordingly, a field for EV engineering has emerged to augment their natural properties (165) and recapitulate their function in semi-synthetic and synthetic EVs. In recent work, Xu et al. introduced a novel peptide-equipped EV platform to enhance the efficiency of EV penetration and oligonucleotide loading capacities (303). More translational examples of the application of EVs to facilitate personalized cancer therapeutic methods were provided by a previous publication (304). Also, the other recent review summarized most of the available examples regarding the administration of native and engineered EVs from various sources, including MSCs, immune, and tissue-specific cells for inflammation therapy in different tissues such as brain, eye, lung, heart, liver, bowel, bone, and skin (181).

## 6 Safety and risk factors associated with cells or EV administration

The administration of cells or EVs should be considered rigorously according to many associated risk factors. All ethical considerations, scientific aspects, and guidelines proposed for cell-based therapies should be considered before EV application (305). In this piece, we try to highlight some issues to sensitize and promote more efforts in evaluating the safety of cells or EV-based therapies.

### 6.1 Contaminations

Both cells and EVs can be contaminated with bioactive components, including but not limited to viruses, bacteria, and endotoxins, that should be checked before any administration. Considering similarities between viruses and exosomes (306–309), viral contaminations for EV preparations are one of the main concerns. Usually, the size of bacteria and fungi are larger than EVs; however, infections specifically by intracellular contaminations such as mycoplasma can change both cell and EVs properties (310–313).

### 6.2 Proliferation

The risk of uncontrolled proliferation of cells is associated with cell-based therapies. Unlike cells, EVs cannot proliferate, and multiple administrations are usually required to reach comparable results. However, more studies are required to show if EVs have machinery like viruses to propagate.

### 6.3 Transfer of genetic materials

Similar to cells, EVs carry DNA (314–316) and mitochondria content (317–319). Recently, it has been shown that genetic materials can also be incorporated into the EV bio-corona (63, 319–321). Therefore, safety concerns are associated with administering both cells and EVs regarding their genetic materials.

### 6.4 Cross-species contaminations

Considering the danger of cross-species contaminations, cells are cultured using human-derived components in xeno-free culture systems. The same issues for EVs should be considered even more rigorously. Firstly, cells can uptake materials from the medium during the culturing process and re-packing them into their EVs derivatives. Second, EVs or particles originating from bovine-derived serum supplements cannot be efficiently removed based on current methods (321–324). Therefore, the risk of cross-species contaminations should be assessed in both cells and EV administration.

### 6.5 Passing through the barriers

Unlike cells, EVs can cross barriers, including but not limited to the blood-brain barrier (325–327). The risk of passing through the barriers is another issue that should be considered explicitly in the EV administration.

The issues raised about the enclosed risk of EV administration belong to unmodified EVs. In cases where there is a need to expose the cells to different physical or chemical agents or to engineer the producer cells or EVs, more details should be taken into account (328). Maybe that is why a debate is being opened about whether exosomes could be considered a medicinal product rather than a biological product similar to their producer cells. The point here remains to be done is the release of internationally accepted guidelines, designed explicitly for EVs and their clinical applications.

## 7 Prospective future: MSC-EVs vs. MSCs

Immunosuppression is among the most favorable therapeutic strategies for managing autoimmune diseases (329–332). Stem cells have been proposed as promising biologicals with superior properties compared to conventional methods (1, 11, 333). Mesenchymal stem cells (MSCs) could be considered one of the safest and most commonly used advanced therapy medicinal products (ATMPs) (334, 335) due to their high regenerative capacities and special immunoregulatory functions (336, 337) affecting all types of innate and adaptive immune cells (338). However, some reviews and meta-analyses questioned why translational outcomes were not as efficient as we expected throughout these years (339). In most cases, short-term effects and the heterogeneous responses of patients to treatment are considered the main challenges. Despite current concerns, MSCs have found their way to treat autoimmune diseases by alleviating the symptoms.

Extensive parallel researches during the last decade has highlighted the importance of MSCs’ paracrine effects (85) and proposed the possibility of replacing the cells with their cell-free products, even in the allogeneic context (147). Here, we debated the potential applications and limitations of extracellular vesicles (EVs) compared to their producer cells, especially in relation to autoimmune diseases. Despite the initial trend, it is now proposed that several challenges and complexities accompany these vesicles (174, 340). Some critical challenges are defining the optimal culture conditions, large-scale production, reproducibility of the isolation procedures, homogenous content of the final prep, efficient and aim-specific functional characterizations, storage, and standardization (148, 341–347). One of the most crucial concerns regarding EVs application is a possible viral infection (348, 349). In addition, viral components can affect EV biogenesis, composition, and secretion of unknown “off-target” side interferences (350).

The other criteria that highlighted the potential of EVs for future therapeutic applications are their ability to be engineered or modified (269, 271, 272, 351, 352). They could be loaded with unexpected target/s or encapsulated with different bio-scaffolds upon the urgent need (237, 353). Furthermore, EVs could be recovered easily following their freeze-thaw cycles (109). These features facilitate their application as a good platform for cellular products in emergencies. Moreover, recent progress in the fields has made their room temperature storage, characterization and maintenance more straightforward and friendly than before, and therefore a significant step closer to their extended application, pharmaceuticalization, and commercialization (163, 354, 355). As it was inferred from the literature, EVs have a broad spectrum of applications. Some applications focus on their ability to pass through blood barriers or other unique properties versus the cells or other synthetic nano-carriers (261, 356). As non-toxic nano-carriers, EVs demonstrated better pharmacokinetics and pharmacodynamics features (absorption, distribution, metabolism, and excretion) than synthetic nano-carriers (140, 141, 357). This may pave the way for application of EVs as the smart carrier for conventional drugs in the context of autoimmune diseases (267).

Although EVs are reported to be efficient in dampening the symptoms of different immunological and non-immunological diseases, sometimes with a completely different mechanism of action (182, 242), they may not seem as efficient as their parent cells. We noted that it is not already possible to assess the exact amount of EVs which are functionally equal and comparable to a specific number of transplanted cells. Nevertheless, EVs have the potential for repeated rounds and off-the-shelf applications. To increase the efficiency of the treatments, in the case of some acute or chronic diseases, including autoimmune disorders, the simultaneous application of cell- and EV-therapy procedures were proposed and are currently under clinical investigation (ClinicalTrials.gov Identifiers: NCT05387278, NCT05520125). It is assumed that the combinatorial strategy may provide the patients with enhanced and prolonged effectiveness of the biological treatments (358, 359). Future research will determine whether EVs are efficient enough or if we still need the cells to reach the proper efficiency.

Regardless of the safety, the central morality of the EV application is of the same importance. The efficiency of the treatments and the cost-benefit aspect of EVs administration for patients is critical when physicians allocate patients to new treatment strategies. Many questions need to be addressed in this regard. Whether this method is applied as an effective treatment or only as a palliative method? Does the patient have enough knowledge and a good idea about this treatment method and its effectiveness? Undoubtedly, having their consent is necessary when participating in a clinical trial.

As we focused on autoimmune diseases in the current piece, we should emphasize the pathogenesis of the disease, the current status of the patients, the disease status, and the availability of strong support from previous pre-clinical and clinical studies are essential before referring the patients. Fortunately, among different diseases and conditions, clinicians are provided with more valid data from pre-clinical studies (360–363), clinical trials (50, 364–366), and comprehensive reviews (257, 367, 368) on the efficiency of EVs in the frame of different autoimmune diseases (369). Although it is inferred from pre-clinical and clinical data that MSC-EVs are safe and efficient (51, 370), and great promise is accompanied by their future applications for treating life-threatening autoimmune diseases, this is of particular importance that we avoid generalization and prejudice regarding their overall efficiency. Other than personalized medicine aspects, it should be considered that each disease has its unique pathogenesis, grading, and mechanisms of spread/progression, which is crucial when we decide to apply EVs for their treatment. Moreover, our current knowledge is not equivalent in the case of different autoimmune diseases regarding the consequences of MSC-EVs administration. Further, although, based on the current data, small extracellular vesicles (sEVs) could be enriched with immunomodulatory components (371, 372), it should be confirmed practically in proper autoimmune disease models and human cases that it has a real functional impact. Despite increasing data that EVs are potent immunomodulators in mimicking their producer cells (146, 207, 373), it is yet to be confirmed whether EVs are superior to their producer cells and, if so, which subtype of EVs is preferred for different autoimmune diseases. Upon defining the GMP-compatible protocols and standardization, the next step will be designing the procedures for preparing modified disease-specific EVs with ideal functional properties (374). These EVs may carry a higher level of immunoregulatory genes, RNAs, miRNAs, lipids, and proteins or target specific molecules, cells, or tissues.

## Author contributions

AH (PhD): Literature review, designing, writing the manuscript, drafting tables and figures, and approval the final version of the manuscript. HB (MD): Conceptualization, drafting, critical editing, and approval the final version of the manuscript. MMa (PhD): Drafting, contribution to design, critical editing, and approval the final version of the manuscript. FS (PhD): Drafting, contribution to design, critical editing, and approval the final version of the manuscript. ME (PhD): Editing, and approval the final version of the manuscript. RL (PhD): Editing, and approval the final version of the manuscript. NA (PhD): Drafting, contribution to design, critical editing, and approval the final version of the manuscript. MMi (PhD candidate): Graphical design of figures, editing, and approval the final version of the manuscript. AB (PhD): Conceptualization, drafting, supervising, funding acquisition, and approval the final version of the manuscript. HB (MD, PhD): Conceptualization, drafting, supervising, funding acquisition, and approval the final version of the manuscript. All authors contributed to the article and approved the submitted version.

## Glossary

**Table:**

(ADWP)	Autoimmune diseases working party
(FDA)	Food and drug administration
(AD)	Adipose tissue-derived
(ALI)	Acute lung injury
(AMI)	Acute myocardial infarction
(APCs)	Antigen-presenting cells
(ARDS)	Acute respiratory distress syndrome
(AREG)	EGFR ligand, amphiregulin
(ATMPs)	Advanced therapy medicinal products
(AU)	Autoimmune uveitis
(BBB)	blood-brain barrier
(BM)	Bone marrow
(BMA)	Bone marrow aspirate
(CD34)	Cluster of differentiation 34
(CD47)	Cluster of differentiation 47
(CD63)	Cluster of differentiation 63
(CD81)	Cluster of differentiation 81
(CD)	Crohn’s disease
(CIMVs)	Cytochalasin B-induced membrane vesicles
(DDS)	Drug delivery system
(EBMT)	European society for blood and marrow transplantation
(ECM)	Extracellular matrix
(EVs)	Extracellular vesicles
(GMP)	Good manufacturing practice
(GvHD)	Graft versus host diseases
(HSC)	Hematopoietic stem cell
(HSC70)	Heat shock protein 70 family
(IBD)	Inflammatory bowel disease
(ISBT)	International society of blood transfusion
(ISCT)	International society for cell and gene therapy
(ISEV)	International society for extracellular vesicles
(ISSCR)	International society for stem cell research
(ITP)	Immune Thrombocytopenia
(MSC)	Mesenchymal stem/stromal cell
(MTF)	Mesenchymal trophic factor
(MS)	Multiple sclerosis
(OA)	Osteoarthritis
(PBMC)	Peripheral blood mononuclear cells
(PPRs)	Pattern recognition receptors
(RA)	Rheumatoid arthritis
(SIMPLE)	Small integral membrane proteins of the lysosome/late endosome
(SjS)	Sjogren’s syndrome
(ST6Gal-1)	β-galactoside α2,6-sialyltransferase 1
(SVF)	Stromal vascular fraction
(SLE)	Systemic lupus erythematosus
(Ti-EVs)	Tissue-derived EVs
(tolDCs)	Tolerogenic dendritic cells
(Tregs)	Regulatory T cells
(UC)	Umbilical cord.
